# Sylvester F. Mixer, M.D., of Buffalo

**Published:** 1883-10

**Authors:** 


SYLVESTER F. MIXER, M.D., OF BUFFALO.—Dr. Mixer died on Sunday, the 16th inst., at the age of sixty-eight. He war born in Morrisville, N. Y., December 27, 1815, and was graduated from the Medical Department of Yale College in 1841, and in that year began the practice of medicine in Buffalo. In 1847 he took the degree of M.D. from the College of Physicians and Surgeons in this city. He was a member of the Medical Society of the State of New York, and of the Buffalo Medical Society, of which he was president in 1852. He was also a member of the Medical Society of the County of Erie, and of the American Medical Association, and from 1858 to 1874 was attending physician to the Buffalo City Hospital, to which institution he was subsequently appointed consulting physician.

---

Dr. Lithgow, in *British Med. Journal*, thus writes of cascara sagrada: "The sacred bark" is from the *rhamnces purshiana*, a tree khown to Æsculapius. At least three species of the rhamnaceæ are known to medicine, viz.: rhamnces catharticus — common buckthorn; rhamnces frrangula—the black alder, and the tree mentioned above.

My attention was first directed to cascara sagrada by an American pamphlet which fell into my hands some years ago, since which time I have prescribed it with almost invariably successful results, and cannot but regard it, under favorable conditions, as a specific in cases of chronic constipation.

